# The Involvment of Hematopoietic-Specific PLC -β2 in Homing and Engraftment of Hematopoietic Stem/Progenitor Cells

**DOI:** 10.1007/s12015-016-9689-x

**Published:** 2016-10-04

**Authors:** Mateusz Adamiak, Malwina Suszynska, Ahmed Abdel-Latif, Ahmed Abdelbaset-Ismail, Janina Ratajczak, Mariusz Z. Ratajczak

**Affiliations:** 1 Stem Cell Institute at James Graham Brown Cancer Center, University of Louisville, 500 South Floyd Street, Louisville, KY 40202 USA; 2 Division of Cardiovascular Medicine, Gill Heart Institute, University of Kentucky, Lexington, KY USA; 3 Department of Regenerative Medicine, Warsaw Medical University, Warsaw, Poland

**Keywords:** PLC-β2, Stem cell homing, HO-1, SDF-1, S1P, C1P

## Abstract

Migration and bone marrow (BM) homing of hematopoietic stem progenitor cells (HSPCs) is regulated by several signaling pathways, and here we provide evidence for the involvement in this process of hematopoietic-specific phospholipase C-β2 (PLC-β2). This enzyme is involved in release of intracellular calcium and activation of protein kinase C (PKC). Recently we reported that PLC-β2 promotes mobilization of HSPCs from BM into peripheral blood (PB), and this effect is mediated by the involvement of PLC-β2 in the release of proteolytic enzymes from granulocytes and its role in disintegration of membrane lipid rafts. Here we report that, besides the role of PLC-β2 in the release of HSPCs from BM niches, PLC-β2 regulates the migration of HSPCs in response to chemotactic gradients of BM homing factors, including SDF-1, S1P, C1P, and ATP. Specifically, HSPCs from PLC-β2-KO mice show impaired homing and engraftment in vivo after transplantation into lethally irradiated mice. This decrease in migration of HSPCs can be explained by impaired calcium release in PLC-β2-KO mice and a high baseline level of heme oxygenase 1 (HO-1), an enzyme that negatively regulates cell migration.

**Electronic supplementary material:**

The online version of this article (doi:10.1007/s12015-016-9689-x) contains supplementary material, which is available to authorized users.

## Introduction

The phospholipase C (PLC) family of enzymes consists of 13 members split between six subfamilies, including the PLC-δ (1, 3, 4), −β (1–4), −γ (1, 2), −ε, −ζ, and –η (1, 2) isoforms [1–3]. PLC enzymes are associated with cell surface receptors that convert phosphatidyloinositol-4,5-biphosphate into two important second messengers, diacylglycerol (DAG) and inositol-1,4.5-triphosphate (IP_3_) [3–5]. Among these isoforms, PLC-β2 is somewhat unique in being a hematopoietic-specific enzyme [1, 2].

Recently, we identified PLC-β2 as the first known lipolytic enzyme involved in the mobilization of hematopoietic stem/progenitor cells (HSPCs) from bone marrow (BM) into peripheral blood (PB) [5, 6]. These pro-mobilizing effects depend on two important mechanisms. First, PLC-β2, as an intracellular enzyme involved in signaling from the receptor for the C5 cleavage fragment C5a (C5aR), promotes degranulation of granulocytes, which release proteolytic enzymes affecting cell adhesion-mediated retention mechanisms of HSPCs in their BM niches. These retention mechanisms involve the chemokine receptor CXCR4 and the very late antigen 4 receptor (VLA-4, also known as α_4_β_1_ integrin) expressed on the surface of HSPCs. Their respective ligands, the α-chemokine stromal cell-derived factor 1 (SDF-1) and vascular adhesion molecule 1 (VCAM-1, also known as CD106), are expressed by cells in the BM microenvironment (e.g., osteoblasts and fibroblasts) [1, 6–11]. Secondly, PLC-β2, when released extracellularly from granulocytes and HSPCs upon stimulation, cleaves the glycolipid glycosylphosphatidylinositol anchor (GPI-A) in cell membranes and thus disrupts the structure of membrane lipid rafts, which are important in the retention of HSPCs in BM niches [5, 6, 12]. It is well known that both BM-retention receptors for HSPCs, CXCR4, and VLA-4, are membrane lipid raft receptors [6, 13–17].

Taking into consideration the important role of PLC-β2 in promoting detachment of HSPCs from BM niches, it is not surprising that PLC-β2-KO mice are poor mobilizers [5]. Nevertheless, while performing mobilization studies, we found that BM cells from these animals show somewhat reduced chemotaxis in response to several chemottractants involved in cell trafficking. Therefore, we became interested in the role of PLC-β2 in regulating the migration of HSPCs, as this enzyme is potentially involved in BM homing of HSPCs after transplantation.

However, in an initial old report describing PLC-β2 knockout mice, PLC-β2 was proposed to inhibit cell migration [1], its contrasting migration-promoting role for T lymphocytes was demonstrated in more recent work [18]. As of today, the overall consensus is that PLC signaling does not inhibit [1] but instead promotes cell trafficking [18].

We report here that HSPCs from PLC-β2-KO mice show defective migration in response to BM-released chemoattractants and as result of this show impaired homing and engraftment in vivo after transplantation into lethally irradiated mice. This decrease in migration of HSPCs can be explained, at least partially, by impaired calcium release and (phosphokinase C) PKC activation in PLC-β2-KO mice and an enhanced intercellular baseline level of the heme oxygenase 1 (HO-1) enzyme, which, as we recently reported, negatively regulates cell migration [19].

## Material and Methods

### Animals

Pathogen-free, 4–6-week-old C57BL/6 J wild-type mice (WT) and B6.129S1-Plcβ2^tm1Dwu^/J (PLC-β2-KO) female mice were purchased from the Jackson Laboratory (Bar Harbor, ME; USA) at least 2 weeks before experiments. Animal studies were approved by the Animal Care and Use Committee of the University of Louisville (Louisville, KY, USA) [5, 20].

### Murine Bone Marrow-Derived Mononuclear Cells (BMMNCs)

BMMNCs were obtained by flushing tibias and femurs from WT and PLC-β2-KO mice. Red blood cells (RBCs) were removed by lysis in BD Pharm Lyse buffer (BD Biosciences, San Jose, CA, USA), washed, and resuspended in appropriate media [21].

### Sorting of Gr-1^+^ Cells

BM was flushed from the femurs and tibias of experimental mice, and after lysis of RBCs using 1 × BD Pharm Lyse buffer (BD Pharmingen, San Jose, CA, USA) the population of total nucleated cells was obtained. Cells were subsequently stained with antibodies: allophycocyanin (APC)–Cy7–anti-Ly6G (clone 1 A8), phycoerythrin (PE)–anti-CD11b (clone M1/70), and Alexa Fluor 488–anti-Ly6C (clone HK1.4) for 30 min in medium containing 2 % FBS (fetal bovine serum). The cells were then washed, resuspended in RPMI 1640 medium, and sorted using a Moflo XDP cell sorter (Beckman Coulter, Indianapolis, IN, USA) as populations of neutrophils (Ly6G^+^/CD11b^+^) [22, 23].

### Transwell Migration Assay

BMMNCs and BM-derived Gr-1^+^ cells from WT and PLC-β2-KO mice were resuspended in assay medium (RPMI-1640 with 0.5 % BSA). Assay medium (650 μl), alone or containing stromal-derived growth factor 1 (SDF-1, 100 ng/ml), sphingosine-1-phosphate (S1P, 0.1 μM), ceramide-1-phosphate (C1P, 100 μM), or adenosine triphosphate (ATP, 0.25 μg/ml) for BMMNCs or SDF-1, chemokine (C-C motif) ligand 5 (CCL5, also known as RANTES, 75 ng/ml), or chemokine (C-C motif) ligand 3 (CCL3, also known as MIP-1α, 10 ng/ml) for BM-derived Gr-1^+^ cells, was added to the lower chambers of a Costar Transwell 24-well plate (Corning Costar, Cambridge, MA, USA). Aliquots of cell suspension (1 × 10^6^ cells per 100 μl) were loaded onto the upper chambers with 5-μm pore filters and then incubated for 3 h (37 °C, 5 % CO_2_). Aliquots of BMMNCs and Gr-1^+^ cells from the lower chambers were harvested and scored by FACS analysis. Briefly, the cells were gated according to their forward-scatter (FSC) and side-scatter (SSC) parameters and counted during a 30-s acquisition at a high flow rate. The rest of the BMMNCs recovered from the lower chamber were resuspended in human methylcellulose base medium provided by the manufacturer (R&D Systems), supplemented with murine GM-CSF (25 ng/ml) and IL-3 (10 ng/ml) for determining the number of CFU-GM colonies. Cultures were incubated for 7 days (37 °C, 95 % humidity, and 5 % CO_2_), at which time they were scored under an inverted microscope for the number of colonies [14, 19, 21].

### Fibronectin Cell-Adhesion Assay

Murine BMMNCs were resuspended in RPMI 1640 plus 0.5 % bovine serum albumin (BSA) medium (5 × 10^4^cells/100 μl). Subsequently, cell suspensions were incubated for 15 min at 37 °C, added directly to 96-well plates coated before the experiment with fibronectin (10 μg/ml), incubated overnight at 4 °C, and then blocked with medium containing 0.5 % BSA for 2 h at 37 °C. Non-adherent cells were then washed from the wells, and all adherent cells were counted using an inverted microscope [14, 19].

### Short-Term Homing Experiments

C57BL/6 J (B6) mice were separated into two groups and irradiated with a lethal dose of γ-irradiation (1000 cGy). Twenty-four hours after irradiation, the animals were transplanted (by tail vein injection) with 4 × 10^6^ BM cells from WT or PLC-β2-KO mice. Before transplantation cells were labeled with PKH67 Green Fluorescent Cell Linker (Sigma-Aldrich, St Louis, MO, USA) according to the manufacturer’s protocol. At 24 h after transplantation, BM cells from the femurs were isolated via Ficoll-Paque and divided. A part of the cells were analyzed on a flow cytometer. The rest of the cells were plated in serum-free methylcellulose cultures and stimulated to grow CFU-GM colonies with granulocyte-macrophage colony-stimulating factor (GM-CSF, 25 ng/ml) and interleukin 3 (IL-3, 10 ng/ml). After 7 days of incubation (37 °C, 95 % humidity, and 5 % CO_2_) the number of colonies was scored under an inverted microscope [19, 20].

### Evaluation of Engraftment

For engraftment experiments, C57BL/6 J (B6) mice were divided into two groups and irradiated with 1000 cGy γ-irradiation (lethal dose). After 24 h, mice were transplanted with 1.5 × 10^5^ BM cells from WT or PLC-β2-KO mice by tail-vein injection. Femora of transplanted mice were flushed with phosphate-buffered saline (PBS) on day 12 post transplantation. Purified via Ficoll-Paque, BM cells were plated in serum-free methylcellulose cultures and stimulated to grow CFU-GM colonies with G-CSF (25 ng/ml) plus IL-3 (10 ng/ml). After 7 days of incubation (37 °C, 95 % humidity, and 5 % CO_2_) the number of colonies was scored under an inverted microscope. Spleens from experimental mice were also removed, fixed in Telesyniczky’s solution for CFU-S assays, and the colonies on the surface of the spleen counted [19, 20].

### Recovery of Leukocytes and Platelets

For transplantation experiments, mice were irradiated with a lethal dose of γ-irradiation (1000 cGy). After 24 h, the mice were transplanted by tail-vein injection with 2.5 × 10^5^ BM cells. Transplanted mice were bled at various intervals from the retro-orbital plexus to obtain samples for white blood cell (WBC) and platelet (PLT) counts as described. Briefly, 50 μl of PB were taken into EDTA-coated Microvette tubes (Sarstedt Inc., Newton, NC, USA) and run within 2 h of collection on a HemaVet 950FS hematology analyzer (Drew Scientific Inc., Oxford, CT, USA) [19, 20].

### Calcium Level

Murine Sca-1^+^ enriched for HSPCs were isolated by immune-magnetic beads and Gr-1^+^ cells sorted as described [5] were resuspended in RPMI 1640 medium supplemented with 0.5 % BSA (2 × 10^6^ cells per 400 μl medium) and incubated overnight at 37 °C. Subsequently, cells were stimulated by adding SDF-1 (100 ng/ml), S1P (0.1 μM), C5a (140 ng/ml), or _desArg_C5a (140 ng/ml) and incubated for 3 h at 37 °C. The cells were then centrifuged, and conditioned media (CM) were collected. Calcium levels were measured using the Calcium Quantification Assay kit (Abcam, Cambridge, MA, USA), according to the manufacturer’s protocol, by measuring the fluorescence in a fluorescence microplate reader (Beckman Coulter DTX 880 Multimode Detector, Beckman Coulter) using excitation at 540 nm and emission detection at 590 nm.

### HO-1 Gene Expression by Real-Time Quantitative Reverse-Transcription PCR

Total RNA from WT and PLC-β2-KO mice was isolated from BM cells using RNeasy Kit (Qiagen, Valencia, CA) according to manufacturer’s protocol and then reverse-transcribed with MultiScribe reverse transcriptase and oligo-dT primers (Applied Biosystems, Foster City, CA). Quantitative assessment of mRNA levels was done by real-time qRT-PCR with Power SYBR Green PCR Master Mix reagent using ABI 7500 instrument. PCR conditions: 95 °C (15 s), 40 cycles at 95 °C (15 s), 60 °C (1 min). Only one PCR product was amplified, according to melting point analysis. The relative quantity of a target, normalized to the endogenous β2-microglobulin gene as control and relative to a calibrator, is expressed as 2^–ΔΔCt^ (fold difference), where Ct is the threshold cycle, ΔCt = (Ct of target genes) – (Ct of the endogenous control gene, β2-microglobulin), and ΔΔCt = (ΔCt of samples for the target gene) – (ΔCt of the calibrator for the target gene) [14, 19]. Primer pairs used for the analysis: 5′-CCT CAC AGA TGG CGT CAC TT-3′ (forward) and 5′-GCT GAT CTG GGG TTT CCC TC-3′ (reverse).

### Western Blotting

To analyze the expression of HO-1, BMMNCs from WT and PLC-β2-deficient mice were harvested, centrifuged, and washed twice with PBS. Protein extracts were then obtained after cell lysis using RIPA lysis buffer supplemented with protease and phosphatase inhibitors (Santa Cruz Biotechnology) and centrifugation at 15,000 rpm at −4 °C for 15 min. The protein concentrations were measured with the Pierce BCA Protein Assay Kit (Pierce, Rockford, IL) and Multimode Analysis Software (Beckman Coulter). Next, adjusted protein lysates (80 μg per sample) were separated on a 4–12 % SDS-PAGE gel, and fractionated proteins were transferred to a PVDF membrane (Bio-Rad). After blocking with 2.5 % non-fat dry milk in Tris-buffered saline containing 0.1 % Tween (TBST) for 1 h at room temperature, then washing with TBST, the membranes were incubated with a rabbit anti-HO-1 polyclonal antibody (Enzo Life Sciences, NY, USA, diluted 1:1000) overnight at 4 °C. Equal loading of proteins in all lanes was assured by reprobing with rabbit anti-β-actin monoclonal antibody (Novus Biologicals, USA, diluted 1:1000). Enhanced chemiluminescence (ECL) reagent (Amersham Life Sciences) and film (Hyperfilm, Amersham Life Sciences) were used for band visualization [19, 21].

### Statistical Analysis

All results are presented as mean ± SD. Statistical analysis of the data was done using Student’s t-test for unpaired samples, with *p* ≤ 0.05 considered significant.

## Results

### Bone Marrow Mononuclear Cells (BMMNCs) from PLC-β2-KO Mice Show an Impaired Chemotactic Response to BM Homing Factors

**Fig. 1:**
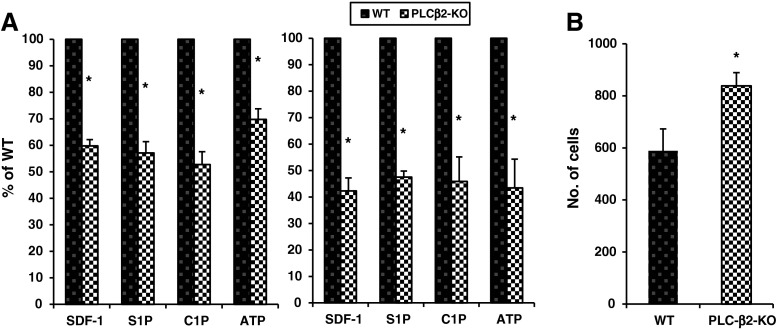
The influence of PLC-β2 on chemotaxis and adhesion of murine BMMNCs. Panel **a** The chemotactic responsiveness to SDF-1, S1P, C1P, and ATP gradients of BMMNCs (left panel) and clonogenic CFU-GM progenitors (right panel) from PLC-β2-KO compared with WT mice. Results are combined from three independent experiments and shown as a percent of results obtained with WT mice.*p > 0.05. Panel **b** The effect of PLC-β2 on adhesion of murine BMMNCs to fibronectin- coated plates. The results are shown as the number of adherent cells. Data from four separate experiments are pooled together. *p < 0.01

It is known that responsiveness to BM-derived chemotactic factors directs homing of HSPCs [10, 24–26]. Therefore, we first evaluated the responsiveness of murine BMMNCs to stromal-derived factor 1 (SDF-1), sphingosine-1-phosphate (S1P), ceramide-1-phosphate (C1P), and adenosine triphosphate (ATP) gradients by employing Transwell migration assays. Figure 1a shows that BMMNCs and more specifically clonogenic CFU-GM cells from PLC-β2-KO mice have impaired chemotaxis to all chemoattractants tested in the current study, which are all known to be involved in the homing of HSPCs to BM [25, 26].

This decrease in chemotaxis was paralleled by increased spontaneous adhesion of BMMNCs to fibronectin-coated plates (Fig. 1b).

### Bone Marrow Cells from PLCβ-2-KO Mice Show Defective Homing and Engraftment after Hematopoietic Transplantation

Since BMMNCs from PLC-β2-KO mice show defective in vitro migration in response to BM-secreted homing-relevant chemoattractants, we next moved to an in vivo model and employed three different assays.

**Fig. 2:**
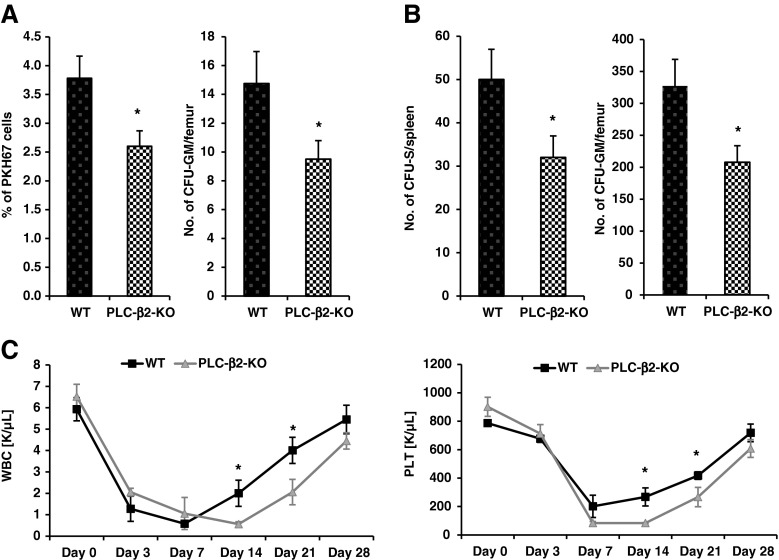
Defects in homing and short- and long-term engraftment of HSPCs from PLC-β2-KO mice. Panel **a** Lethally irradiated C57BL/6 J mice (six mice per group) were transplanted with 4 × 10^6^ bone marrow mononuclear cells (BMMNCs) from PLC-β2-KO mice and labeled with a PKH67 cell linker with WT as control. Twenty-four hours after transplantation, femoral BMMNCs were harvested, the number of PKH67 cells was evaluated by FACS (panel **a**, left), and the clonogenic CFU-GM progenitors were enumerated in an in vitro colony assay (Panel **a**, right). No colonies were formed in lethally irradiated or untransplanted mice (irradiation control). Panel **b** Lethally irradiated WT mice (six per group) were transplanted with 1.5 × 10^5^ BMMNCs from PLC-β2-KO and WT mice. Twelve days after transplantation the spleens were removed, and femoral BMMNCs were harvested for counting the number of CFU-S colonies (panel **b**, left) and plating to count the number of CFU-GM colonies (panel **b**, right). Panel **c** Lethally irradiated WT mice were transplanted with 2.5 × 10^5^ BMMNCs from PLC-β2-KO and WT cells. White blood cells (Panel **c**, left) and platelets (Panel **c**, right) were counted at intervals (at 0, 3, 7, 14, 21, and 28 days). The data in Panels **a**–**c** represent the combined results from two independent experiments (*n* = 10) for each panel. *p < 0.005

First, we labeled BMMNCs from PLC-β2-KO and WT animals with the cell membrane-binding fluorochrome PKH67 and injected them into lethally irradiated syngeneic WT animals. Figure 2a shows that, 24 h after transplantation, cells from PLC-β2-KO mice homed to BM in lower numbers than WT cells. Next, we transplanted lethally irradiated syngeneic animals with BMMNCs from mutant and WT mice, and 12 days later we evaluated the number of spleen colonies formed by the early progenitor cells known as colony-forming units in spleen (CFU-S) as well as the number of clonogenic CFU-GM progenitors present in the BM of transplanted animals. Figure 2b again shows a decrease in early engraftment of PLC-β2-KO mouse-derived HSPCs. Finally, we analyzed the recovery of peripheral blood counts in lethally irradiated mice transplanted with PLC-β2 and WT cells and observed a delayed recovery of leucocytes and platelet counts (Fig. 2 **c**) in mice transplanted with HSPCs from PLC-β2-deficient mice.

### Defective Homing and Engraftment of Mutant HSPCS Is Explained by Changes in PLC-β2-Mediated Signaling

**Fig. 3:**
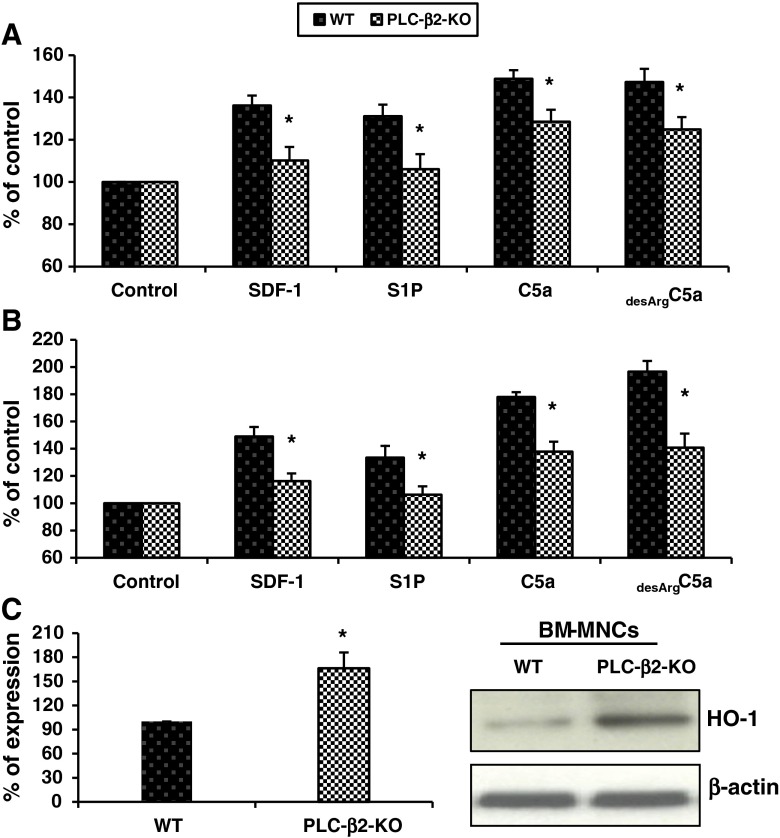
Impact of PLC-β2 on the level of calcium and HO-1 signaling. Panel **a** Calcium level in response to SDF-1, S1P, C5a, and _desArg_C5a was measured in conditioned media from Sca-1^+^ cells enriched for HSPCs (Panel **a**) and Gr-1^+^ cell (Panel **b**) sorted from the BM of PLC-β2-KO and WT mice (as control). Data from two separate experiments are pooled together and compared as the percentage of control. *p < 0.05. Panel **c**, left HO-1 expression was evaluated at the mRNA level by real-time PCR. Results from three independent experiments are pooled together and shown as the percentage of the expression in WT mice. *p < 0.005. Panel **c**, right Expression of HO-1 as detected by western blotting in BMMNCs collected from WT and PLC-β2-KO mice. As shown here, the cells from PLC-β2-KO mice exhibit upregulation of HO-1 compared with WT-derived cells

In our previous paper we already demonstrated that BM-derived cells from PLC-β2 mutant mice have impaired phosphorylation of p42/44 MAPK and AKT after stimulation with mobilization-promoting factors [5]. Therefore, since PLC-β2 is involved in the release of calcium from intracellular stores, we evaluated the intracellular level of Ca^2+^ in BM-sorted Sca-1^+^ cells enriched for HSPCs (Fig. 3a) or Gr-1+ cells (Fig. 3b) from mutant and WT mice in response to the crucial homing factors SDF-1 and S1P. As a positive control, we also stimulated these cells with C5a and _desArg_C5a, which are known to be potent stimulatory factors for these cells. As expected, we observed impaired Ca^2+^ release from the mutant cells.

Finally, since we recently reported that the chemotaxis of HSPCs is negatively regulated by heme oxygenase 1 (HO-1) [19], we evaluated the baseline level of HO-1 in WT and PLC-β2-KO BMMNC cells by RQ-PCR and western blotting (Fig. 3b) and found elevated HO-1 levels in mutant cells, which correlated with their impaired migratory potential.

## Discussion

The most important observation of this report is that hematopoietic-specific PLC-β2 enhances the responsiveness of HSPCs to BM-homing-related chemoattractants, including SDF-1, S1P, C1P, and ATP. By employing BM cells from PLC-β2-KO mice, we provided additional in vivo evidence for the role of this enzyme in directing homing and engraftment of HSPCs.

**Fig. 4:**
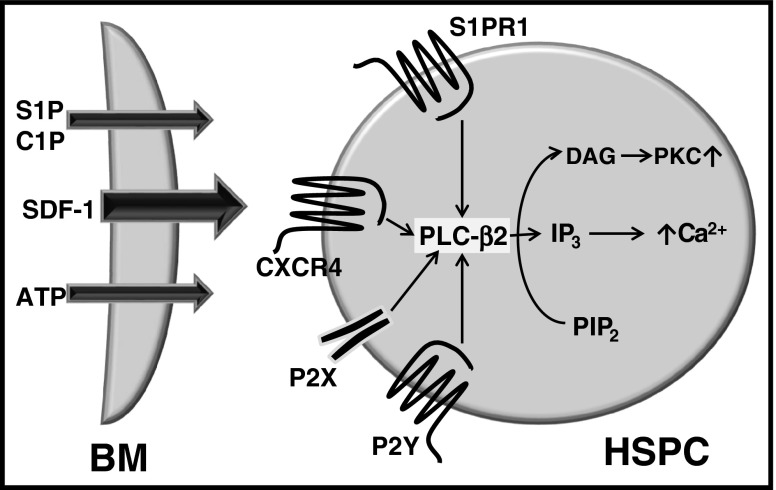
The role of PLC-β2 in homing of HSPCs. PLC-β2 is involved in signaling form all major homing receptors including CXCR4 (SDF-1 receptor), S1PR_1_ (S1P receptor 1), purinergic P2X and P2Y (ATP receptors) and putative receptor for C1P (not cloned yet). Activation of PLC-β2 signaling is crucial for migration and adhesion of HSPCs in DAG – PI3K and IP-3 – Ca^2+^ depended manner

Members of the PLC family play an important role in inducing two second messengers, diacylglycerol (DAG) and inositol three phosphate (IP_3_), in cells [5] (Fig. 4). These second messengers play an important role in activating phosphokinase C (PKC) and in the release of calcium from intracellular stores. It is known that the hematopoietic cell-specific isoform PLC-β2 is associated with several G protein-coupled receptors, including CXCR4, S1PR_1_, and P2Y receptors, which bind HSPC chemoattractants such as SDF-1, S1P, and ATP, respectively [24, 27]. In this migration process, an important role is played by the intracellular Ca^2+^ level as well as by the activation of several downstream kinases, including p42/44 MAPK, AKT, and PKC-ζ [28]. In particular, PKC-ζ has been implicated in an elegant paper on SDF-1–CXCR4-mediated trafficking of human CD34^+^ HSPCs [29]. Moreover, in the same work injection of mice with inhibitory PKC-ζ pseudosubstrate peptides resulted in mobilization of HSPCs from BM into PB [29].

In our previous work we reported that PLC-β2-KO mice are poor mobilizers and explained this phenomenon by positing a requirement for this enzyme in the generation of a proteolytic and lipolytic microenvironment in BM following the degranulation of granulocytes. This microenvironment is responsible for the disintegration of adhesion interactions as well as integrity of membrane lipid rafts on the surface of HSPCs, which are important for BM-retention of these cells [5]. Here we asked: what is the role of PLC-β2 in homing and engraftment of HSPCs after transplantation?

In our experiments we employed, in addition to SDF-1 [29], other chemotactic factors involved in HSPC homing, such as S1P, C1P, and extracellular ATP, and provided the first evidence that this enzyme regulates chemotactic responsiveness not only to SDF-1 but also other BM homing factors. We found that HSPCs from PLC-β2 mice have defective in vitro migration in response to BM homing factors in Transwell assays and in vivo show defective both homing and engraftment if transplanted into syngeneic WT animals.

Since we already reported that PLC-β2 is involved in phosphorylation of p42/44 MAPK and AKT in murine BM cells [5], in the current work we studied its effect on Ca^2+^ release and confirmed that stimulation of murine BM cells, not only by SDF-1 but also by S1P, C1P, and ATP, leads to increases in its intracellular level. It is well known that the intracellular Ca^2+^ level is regulated both spatially and temporally in cells and affects cell polarization, protrusion, retraction, and adhesion at the right place and time required for effective migration of HSPCs [30, 31]. This PLC-β2 effect is, as mentioned above, mediated in cells after activation of G protein-coupled receptors by specific ligands [3, 27].

Another interesting observation is the elevated steady-state level of HO-1 in PLC-β2-KO cells. As we have reported [19], HO-1 is a negative regulator of cell migration and, based on the results reported in this paper, its level may be regulated by PLC-β2. In support of this possibility, HSPCs from mutant animals showed impaired migratory responsiveness to several chemotactic factors. These effects correlate with a decrease in the release of calcium from intracellular stores, a decrease in phosphorylation of AKT and p42/44 MAPK [5], and an increase in HO-1 expression.

Our results reported here also have important implications for inhibiting cancer metastasis, e.g., to BM. Based on our results we argue that inhibition of PLC by specific inhibitors may decrease the migration of cancer cells and thus their metastatic potential. In fact, small molecule inhibitors of this enzyme have already been developed and tested in in vivo animal models [32, 33].

Moreover, based on our previously published results, HSPCs from PLC-β2-KO mice should have more stable membrane lipid rafts [5]. This would explain their enhanced adhesion to fibronectin-coated plates compared with cells from WT control animals. Lipid rafts, however, also play an important role in cell migration by facilitating better contact of the G protein-coupled cell surface receptors CXCR4 and VLA-4 with downstream signaling molecules [5, 6, 12]. In the case of PLC-β2-KO HSPCs, it seems that their pro-adhesive properties are less affected than their pro-chemotactic responsiveness, because, in contrast to adhesion, chemotaxis in these cells is significantly impaired.

Interestingly, in an initial paper published two decades ago that described PLC-β2-KO mice, it was reported that BM cells from these animals had enhanced chemotaxis in response to chemokines, including RANTES and IL-8 [1], which suggested an inhibitory effect of this enzyme on cell trafficking that somewhat contradicted our results. Therefore, we evaluated the responsiveness of BMMNCs to RANTES and IL-8 gradients (**Supplementary Figure** 1) and found that, like the other chemoattractants tested in our study, BMMNCs showed impaired migration in response to these chemokines. Moreover, a defect in migration in PLC-β2-KO cells was observed for other chemotactic factors as well. In corroboration of our observations, PLC-β2 has been confirmed to be required for proper migration of T cells in other more recent papers [18].

In conclusion, we demonstrated for the first time that PLC-β2 signaling is required for proper migratory responsiveness to all known BM-homing chemotactic factors and plays a role in the engraftment of HSPCs. Since in our in vivo hematopoietic reconstitution studies (Fig. 2) we evaluated short term repopulating hematopoietic stem cells (HSCs), in a future serial transplants experiments are needed to assess the compartment of more primitive long tem repopulating HSCs. At the molecular level, this defect described herein in PLC-β2-KO HSPCs depends on the impaired activation of signaling pathways involved in stem cell migration and adhesion and correlates with elevated baseline levels of heme oxygenase 1 (HO-1), an enzyme that is a negative regulator of cell migration [19]. However, since wild type mice still engraft with BMMNC from PLC-β2-KO our data indicates that there are some PLC-β2-independent redundant mechanism that govern homing and engraftment. This is most likely explained by a fact that trafficking of HSPCs, as a developmental ancient physiological mechanism, has to be well protected from potential single gene mutation.

## Electronic supplementary material

Supplementary Figure 1 The chemotactic responsiveness of BMMNCs (**left**) and Gr-1^+^ cells (**right**) from PLC-β2-KO mice to SDF-1, RANTES, and MIP-1α compared with the analogous cells from WT mice. Results are combined from three independent experiments and shown as a percent of migration of cells from WT mice*p > 0.05. (PPTX 95 kb)
