# Supplementary material for: The Involvment of Hematopoietic-Specific PLC -β2 in Homing and Engraftment of Hematopoietic Stem/Progenitor Cells
Source: Stem Cell Rev. 2016 Oct 4;12(6):613–20. doi: 10.1007/s12015-016-9689-x (PMC5106505; doi:10.1007/s12015-016-9689-x)
Supplement: Supplementary file 1 — The chemotactic responsiveness of BMMNCs (left) and Gr-1+ cells (right) from PLC-β2-KO mice to SDF-1, RANTES, and MIP-1α compared with the analogous cells from WT mice. Results are combined from three independent experiments and shown as a percent of migration of cells from WT mice*p > 0.05. (PPTX 95 kb) [file 12015_2016_9689_MOESM1_ESM.pptx]

## Slide 1
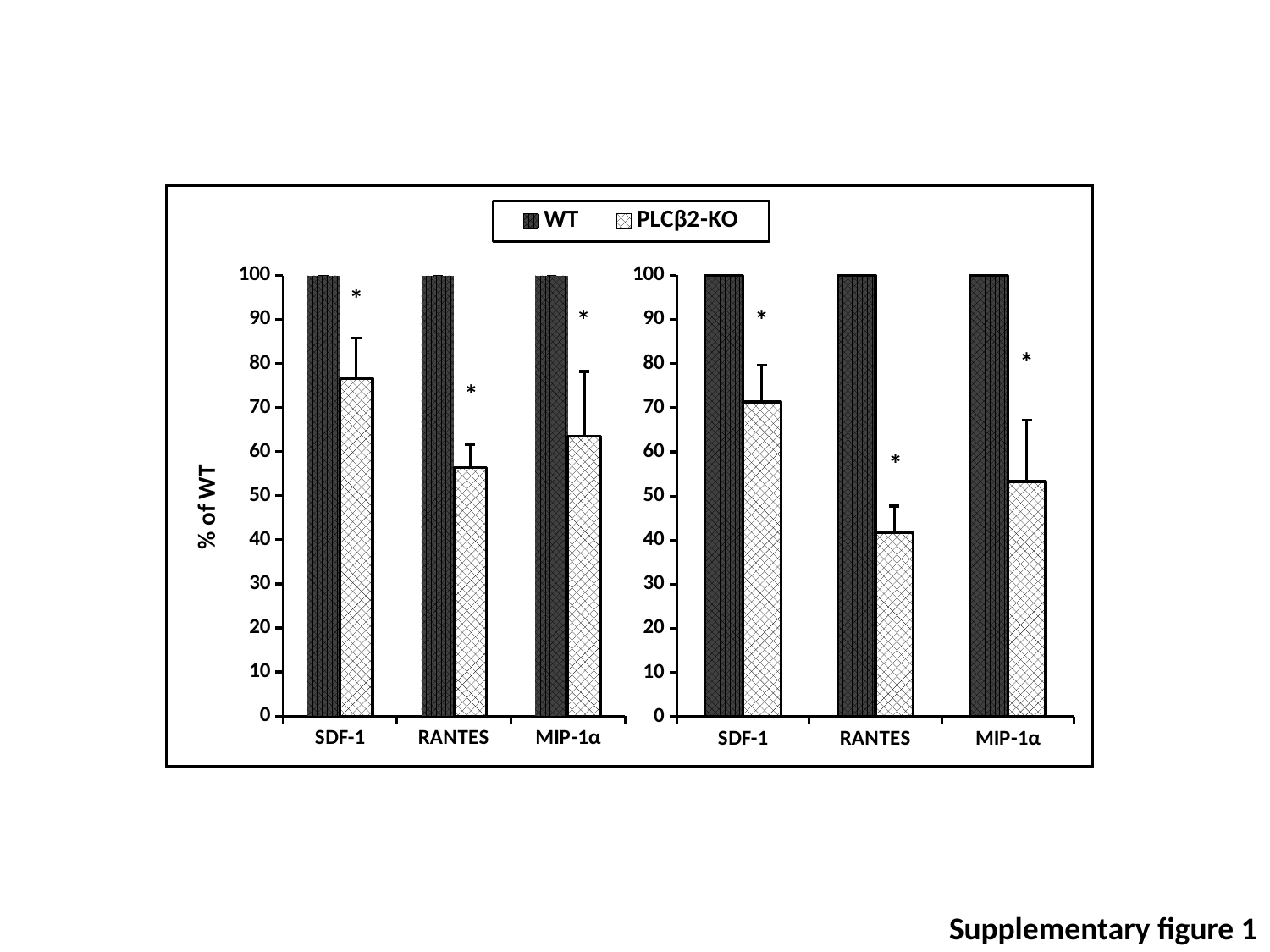

### Chart
| Category | WT | PLCβ2-KO |
|---|---|---|
| SDF-1 | 100.0 | 71.32374451462945 |
| RANTES | 100.0 | 41.61616673558693 |
| MIP-1α | 100.0 | 53.27243390730122 |
### Chart
| Category | WT | PLCβ2-KO |
|---|---|---|
| SDF-1 | 100.0 | 76.61271944131084 |
| RANTES | 100.0 | 56.44793708695856 |
| MIP-1α | 100.0 | 63.58602734864418 |*
*
*
*
*
*
Supplementary figure 1
